# Linguistic features and psychological states: A machine-learning based approach

**DOI:** 10.3389/fpsyg.2022.955850

**Published:** 2022-07-22

**Authors:** Xiaowei Du, Yunmei Sun

**Affiliations:** Department of Foreign Language, Huazhong University of Science and Technology, Wuhan, China

**Keywords:** psychological states, linguistic features, machine learning algorithms, classification, mental disorders

## Abstract

Previous research mostly used simplistic measures and limited linguistic features (e.g., personal pronouns, absolutist words, and sentiment words) in a text to identify its author’s psychological states. In this study, we proposed using additional linguistic features, that is, sentiments polarities and emotions, to classify texts of various psychological states. A large dataset of forum posts including texts of anxiety, depression, suicide ideation, and normal states were experimented with machine-learning algorithms. The results showed that the proposed linguistic features with machine-learning algorithms, namely Support Vector Machine and Deep Learning achieved a high level of performance in the detection of psychological state. The study represents one of the first attempts that uses sentiment polarities and emotions to detect texts of psychological states, and the findings may contribute to our understanding of how accuracy may be enhanced in the detection of various psychological states. Significance and suggestions of the study are also offered.

## Introduction

The language pertinent to mental health has recently emerged as an area of particular interest (Sun et al., 2020). The main rationale of such line of research is that an individual’s psychological state impacts the language used to represent his/her emotions, feelings, and thoughts (Wolohan et al., 2018; Scourfield et al., 2019). These studies may complement previous studies and facilitate the identification of psychological states.

Previous studies have analyzed the linguistic features of texts composed by individuals with psychological issues. The first line of research is the linguistic features that characterize what people with different psychological states are interested in and experiencing (Tadesse et al., 2019; Jones et al., 2020). The second line of research is the linguistic features that reveal how people with different psychological states discuss their interests and experiences (Ji et al., 2018; Boukil et al., 2019).

However, previous studies may be limited in that they used only simplistic indices such as the frequency of sentiment words as the linguistic features (Liehr et al., 2002; Ji et al., 2018). For example, most such studies were performed based on tools such as the Linguistic Inquiry and Word Count Program (LIWC) (Barnes et al., 2007; Lyons et al., 2018; Jones et al., 2020). The LIWC, a widely used commercial tool, covers over 70 dimensions of linguistic features with a lexicon of 2,290 words and word stems (Rude et al., 2004; Sloan, 2005). However, its sentiment module contains only 262 positive words and 345 negative words (Rude et al., 2004; Kahn et al., 2007), while that of its latest version (2015) contains 620 positive words and 744 negative words (Pennebaker et al., 2015). Other studies calculated the frequency of sentiment words based on lexicons such as Ekman-Liberman dictionary (unpublished manual) and Bing (Hu and Liu, 2004). For example, Lieberman and Goldstein (2006) used Ekman-Liberman dictionary which contains 463 negative words, to assess depression severity of breast cancer patients. Mostafa (2013) used Bing (Hu and Liu, 2004), which includes 2,006 positive words and 4,783 negative words, to explore consumer brand sentiments. Another example is Tsugawa et al. (2015) that used a self-developed lexicon of 760 positive words and 862 negative words to recognize depression from Twitter. That is, such tools or methods may not be robust enough to accurately detect psychological states due to not only their limitation in the small number of sentiment words used and emotion types but also the simplistic measure of the frequency of such words (Pennebaker et al., 2003; Kahn et al., 2007; Berkout et al., 2020). As Tausczik and Pennebaker (2010) noted, tools such as the LIWC might incompletely and incorrectly classify words since they cannot recognize the subtle forms of sentiment expression or multiple meanings of words. In addition, most studies only included sentiment polarities such as positive and negative, but they did not consider other sentiment-related dimensions such as emotions of joy, anticipation, disgust, or fear (Sloan, 2005; Kahn et al., 2007; Ziemer and Korkmaz, 2017). More importantly, the method of counting sentiment or emotion words did not consider the strength or intensity of sentiments and emotions (Taboada et al., 2011).

The present study aims to explore the relation between linguistic features and psychological states. To be specific, we employed, in this study, more sophisticated algorithms to analyze the strength or intensity of sentiments and emotions with larger lexicons to detect psychological states. In addition to linguistic features used in previous research such as absolutist words and personal pronouns, sentiments and emotions are also included in the analysis. Meanwhile, we also applied machine learning algorithms in order to improve detection performance. The findings of this study may complement previous studies and facilitate the identification of mental disorders.

## Linguistic features and psychological states

In this section, we review the linguistic features that have been used to recognize psychological states and previous studies that are pertinent to the examination of psychological states via linguistic features.

### Personal pronouns and absolutist words

Linguistic features such as personal pronouns and absolutist words have recently been used to study psychological states.

First, the use of personal pronouns, revealing individuals’ identity focus, is related to individual’s psychological states (Pulverman et al., 2015). To be specific, the use of first-person singular pronouns represents self-focus in that it refers to “self” or “ego” (Newman et al., 2003; Brockmeyer et al., 2015). An excessive and rigid self-focus reflects a lower dominance and higher degree of selfishness, emotional distancing, and social-isolation (Pennebaker et al., 2003; Demiray and Gençöz, 2018), which may increase mental health-related problems such as grief, depression, and suicide ideation (Brockmeyer et al., 2015; Eichstaedt et al., 2018; Allgood et al., 2020). While, the use of other personal pronoun reflects other-focus since it highlights identity focus outward (Tausczik and Pennebaker, 2010). A higher level of other-focus, representing an improvement of social engagement, collectivism, inclusiveness, and group cohesion (Simmons et al., 2008), is related with psychiatric symptom reduction (Cohn et al., 2004; Brockmeyer et al., 2015).

Second, the role of absolutist words (e.g., always, complete, completely) signals a sense of absolutist thinking and effective in identifying mental disorders (Al-Mosaiwi and Johnstone, 2018). Absolutist thinking, as defined by Ostell (1992), is a categorical and evaluative thinking style related to cognitive distortion and irrational beliefs (Savekar et al., 2019). Specifically, it reflects greater certainty, extreme and rigid insistence, and dichotomous thinking in the way people articulate their beliefs (Jones et al., 2020). In other words, it describes magnitudes or probabilities without any form of gradation (Adam-Troian and Arciszewski, 2020). Empirical studies have revealed that the absolutist thinking may cause difficulty in problem-solving, promote dysfunctional emotional states, and do harm to mental health (Jones et al., 2020). For example, absolutist people are less pleasant in their job experience due to their perfectionism (Savekar et al., 2019). Besides, absolutist people are prone to victimization, self-blame, and anger when being criticized or opposed (Jones et al., 2020). In addition, early studies found that suicidal individuals perform more absolutist thinking in response to the concepts such as life and death than that of non-suicidal individuals (Weishaar and Beck, 2009).

### Sentiment and emotion analyses

Sentiment analysis can also be used to identify psychological states. The main reason is that it extracts the polarity of sentiments, attitudes, opinions, and emotions that reveal how people are experiencing the world and what they are anticipating (Liu and Lei, 2018; Rendalkar and Chandankhede, 2018; Zucco et al., 2020). In the narrow sense, sentiment analysis refers to the identification of sentiment polarities, which includes positive, negative, or neutral (Zucco et al., 2020), while in the broad sense, sentiment analysis covers two dimensions, i.e., sentiment and emotion analyses, which allow a more comprehensive identification of sentiments and emotions (Ciullo et al., 2016). Emotion analysis, as one strand of sentiment analysis research, focuses on recognizing a set of basic emotions such as anger, anticipation, disgust, and fear, etc. (Cambria, 2016).

Previous studies have used sentiment analysis, mainly from the perspectives of positive and negative polarities, to identify psychological states (Papapicco and Mininni, 2020b). However, they yielded mixed findings regarding the relation between sentiments and psychological states. On one hand, positive sentiment is positively related to the mental health, and negative sentiment is negatively related to mental health (Pennebaker et al., 2003). For example, Kahn et al. (2007) found that the trend of more negative sentiment words and fewer positive sentiment words may reflect a less healthy mental state. It is worth noting that some studies stressed the impact of negative sentiment expression on psychological states in that negative sentiment words may carry more information of mental health than that of positive sentiment words (Garcia et al., 2012). For example, Herbert et al. (2019) found that people, before committing suicide, use more negative sentiment words in their notes, but no significant change was found in the use of positive sentiment words. On the other hand, some research (e.g., Stone and Pennebaker, 2004) has yielded contradictory findings. Contrary to Stone and Pennebaker (2004) and Herbert et al. (2019) found that a student committing suicide used fewer negative words and more positive words since her mood might have temporarily improved before she committed suicide.

A few of the previous studies used emotion analysis to explore psychological states since emotion affects and reflects individuals’ states of mind (Ciullo et al., 2016). For example, the use of joy or happiness words, revealing a sense of enjoyment, satisfaction, and pleasure, and these words are frequently used when an individual is in the situation of well-being, inner peace, love, safety, and contentment (Papapicco and Mininni, 2020b). Additionally, the use of sadness words reflects the degree of social withdrawal or mood flattening, occurring with a higher frequency when an individual is most likely in grief, loss, frustration, depression, and suicide ideation (Barnes et al., 2007; Eichstaedt et al., 2018; Kim et al., 2019). Another example is De Choudhury et al. (2013), which used sentiments and emotions such as positive, negative, activation, and dominance to detect mothers at risk of postpartum depression, and achieved 71.21% accuracy of detection.

Although previous studies have contributed significantly to our understanding of the relation between linguistic features and psychological states, they may be limited in the linguistic features and the data used in the studies as follows. First, concerning the linguistic features used, many only employed sentiment polarities such as positive and negative (Sloan, 2005; Ziemer and Korkmaz, 2017), and the others used only simplistic indices such as the frequency of sentiment words (Taboada et al., 2011; Ji et al., 2018; Lyons et al., 2018). In addition, most studies used a lexicon of a limited number of sentiment words (Pennebaker et al., 2003; Schwartz et al., 2014). For example, Nguyen et al. (2014) and Herbert et al. (2019) used the lexicon of positive and negative words integrated in the LIWC, which contains, for each category of sentiments, only several hundred words (Pennebaker et al., 2015). Second, the data used in the previous studies seemed limited in size. For example, due to privacy issue, many analyzed only a small sample of notes, letters, diaries, or questionnaires (Desmet and Hoste, 2013; Kim et al., 2019), which may be challenged for its generalizability. It should be noted that recent studies have begun to employ large samples of data collected from social media such as Facebook or Twitter (Schwartz et al., 2014; Tsugawa et al., 2015; Eichstaedt et al., 2018). However, social media data such as tweets may be limited in the amount of information provided since each tweet is less than 280 characters in length (140 characters before 2017) (Papapicco and Mininni, 2020a). Meanwhile, Facebook posts may be challenged regarding the accuracy or truthfulness of their information since they are open to friends and family members (Calvo et al., 2017).

To address the foregoing possible limitations, the present study aims to examine the relation between linguistic features and psychological states by employing an enhanced methodology, and it differs from the previous studies as follows. First, the study used a more comprehensive set of linguistic features. It included not only sentiment polarities, but also eight dimensions of emotions, absolutist words, and personal pronouns. Second, the study used larger lexicons of both sentiment and emotion words (with more than 10,000 words). The use of more comprehensive linguistic features and larger lexicons should provide more accurate measures of psychological states. Third, a large dataset of internet forum posts was used, which were composed of post texts of no word limit. More importantly, the forum posts included texts of several psychological states such as anxiety, depression, and suicide ideation, which should provide us a chance to experiment and detect more variety of psychological states with our proposed linguistic features. Last, more sophisticated techniques such as machine learning algorithms were employed to classify texts by authors with different psychological states. We hope that, with our enhanced methodology, we can build on previous studies to make a contribution to the understanding of the relations between linguistic features and mental health. Specifically, based on the foregoing discussion, the present study aims to address the following research questions.

Research question 1: Do the measures of each of the linguistic features (i.e., absolutist words, first-person pronouns, sentiment polarities, and emotions) vary across the four psychological states, namely normal condition, anxiety, depression, and suicide ideation?

Research question 2: How accurately can the linguistic features classify the texts of four different psychological states?

## Materials and methods

In this section, we introduce the data and the methods for the text analysis and the classification tasks in this study.

### Data

The data used in the present study were a set of internet forum posts collected with rigorous criteria such as word limit, authors, and prose (see Al-Mosaiwi and Johnstone, 2018, for a detailed description of the dataset). We used Al-Mosaiwi and Johnstone (2018) dataset in the study for the following reasons. First, the data consisted of texts from social media such as forum posts. Research suggests that social media data not only provide a large and authentic dataset for the study of mental health but also are rich in information of psychological states (Gilgur and Ramirez-Marquez, 2020). Second, the data contained forum texts of different psychological states as well as control texts (i.e., texts collected from general forums). Therefore, the data were suitable for identifying the relation between linguistic features and different psychological states. We used four groups of forum posts for the experiments in this study: general, anxiety, depression, and suicide ideation. A summary of the data used in this study is presented in Table 1.

**TABLE 1:** A summary of the data.

Groups	Post numbers	Word counts
General	1,050	223,495
Anxiety	614	221,687
Depression	554	206,488
Suicide ideation	327	132,340

### Linguistic features and text analysis

As previously discussed, we employed various linguistic features closely related to mental health to classify texts in the four groups of forum posts. The linguistic features included absolutist words, first-person pronouns, sentiments, and emotions as summarized in Table 2.

**TABLE 2:** Linguistic features.

Categories	Descriptions
Absolutist words	*Absolutely, always, complete, completely, constant, constantly, whole, all, definitely, entire, ever, every, everyone, everything, full, must, never, nothing, totally*
Personal pronouns	First-person singular pronouns (*i, my, me*) First-person plural pronouns (*we, our, us*)
Sentiments	Sentiment polarities
Emotions	Emotions of anger, anticipation, disgust, fear, joy, sadness, surprise, and trust

We included the 19 absolutist words used in Al-Mosaiwi and Johnstone (2018) and the six first-person pronouns used in Tausczik and Pennebaker (2010) in our study. Procedurally, we first calculated the total frequency of the 19 absolutist words and that of the six personal pronouns of each post. The calculations were performed with a self-written Python script. Then, we normalized the raw frequency of the absolutist words and personal pronouns to eliminate the impact of different post lengths (see Formula 1).


(1) $$N\,o\,r\,m\,a\,l\,i\,z\,e\,d\,f\,r\,e\,q\,u\,e\,n\,c\,y=\frac{R\,a\,w\,f\,r\,e\,q\,u\,e\,n\,c\,y}{N\,u\,m\,b\,e\,r\,o\,f\,w\,o\,r\,d\,s\,i\,n\,t\,h\,e\,p\,o\,s\,t}\times 1000$$

In addition, we calculated the values of sentiment and emotions of each text with Rinker (2019) *sentimentr* in R (version 3.6.0). It is worth noting that Rinker (2019) *sentimentr* may outperform others in making the results more reliable with the following reasons. First, it could calculate the sentiment and emotion of each text based on the mean sentiment and emotion values at the sentence level (Rinker, 2019). Second, it covers the relatively comprehensive and widely used lexica to employ sentiment and emotion analysis, respectively (Rinker, 2018). For example, we chose the combined lexicon from Hu and Liu (2004) and Jockers (2017), namely, *Jockers_rinker* to calculate sentiment values since it contains 11,710 sentiment words. Meanwhile, we used the *Jockers* lexicon (Jockers, 2017) to calculate emotion values since it could assign more emotion types with 10,738 words, i.e., anger, anticipation, disgust, fear, joy, sadness, surprise, and trust. More importantly, Rinker (2019) *sentimentr* is an augmented package since it considers valence shifters such as negators, amplifiers (intensifiers), de-amplifiers (downtoners), and adversative conjunctions (Rinker, 2021).

Last, we scaled the normalized frequency of the absolutist words and personal pronouns and the values of sentiment and emotions with a homemade R script for the follow-up statistical analysis and classification tasks.

### Statistical analysis and classification algorithms

First, we performed the Kruskal–Wallis tests to examine if any significant difference existed in the use of the linguistic features across the four groups of texts. Then, we performed the classification tasks based on machine-learning algorithms with the *RapidMiner Studio* (the educational version 9.7). We used machine-learning algorithms since they can automatically and efficiently perform classification tasks with fairly accurate results (Ji et al., 2018). More specifically, we adopted the five machine learning algorithms integrated in the *RapidMiner Studio* for the classification tasks, that is, Naïve Bayes, Generalized Linear Model, Logistic Regression, Deep Learning, and Support Vector Machine. We did so for the following reasons. First, Naïve Bayes, Logistic Regression, and Support Vector Machine are three classic but popular machine learning algorithms for detecting psychological states (Tadesse et al., 2019). Second, Generalized Linear Model and Deep Learning are state-of-the-art and powerful algorithms widely used in recent research (Arora et al., 2016; Nykodym et al., 2020). To better understand the machine learning algorithms we used, we briefly summarized their definitions as follows.

Naïve Bayes (NB) is a supervised learning probabilistic classifier rooted in a robust statistical foundation (Tadesse et al., 2019). It is simple, fast, and accurate in calculation, even in noisy or missing data situations (Kotu and Deshpande, 2015). However, it cannot learn interactions between features since it assumes that each feature is independent and equally important (Nguyen et al., 2017).

Generalized Linear Model (GLM) is an extension of traditional linear models. More specifically, this algorithm fits generalized linear models to the data by maximizing the log-likelihood (RapidMiner, 2022). It can scale well with large datasets based on its flexible structure (Nykodym et al., 2020).

Logistic Regression (LR) is a non-regularized logistic regression that combines the logistic and linear models (Nykodym et al., 2020). Notably, it performs well for binary or binomial classification where the target variable is a categorical variable with two levels (Nguyen et al., 2017). However, it may not be intuitive when dealing with several predictors (Kotu and Deshpande, 2015).

Deep Learning works by a multi-layer feed-forward artificial neural network (Nadeem et al., 2016). It performs well at modeling nonlinear relationships (Paul et al., 2019). However, it requires pre-processing data, takes more time to train, and does not easily explain the inner workings (Wang et al., 2020).

Support Vector Machine (SVM) is a boundary detection algorithm that fixes a hyperplane separating data points into two different classes (Elarnaoty and Farghaly, 2018). It tends to be robust but performs slowly with big data and is only used for binary classification (Desmet and Hoste, 2013).

The classification task was performed as follows. First, we input the data into Machine learning algorithms. More specifically, we directly input the data of linguistic features, namely, the scaled normalized frequency of personal pronouns and absolutist words and the values of sentiment and emotion, since they were calculated into a numeric format. Besides, the mental states, as categorical data types, are variables treated as just names. Second, we split the data into the training and testing sets in terms of the default setting (60/40-ratio) in RapidMiner Studio. In other words, the 60% partition will become the training set we build our model. The remaining 40% will become the test set against which we can compare our model’s predictions. It is worth noting that many studies (e.g., Moustafa and Slay, 2016) have adopted such a ratio and confirmed its effectiveness. Third, we used the four categories of linguistic features and their combinations to distinguish healthy controls (texts of general or healthy states) from mental disorders (texts of anxiety, depression, and suicide ideation). Fourth, we evaluated the results or performance of the classification models with criteria such as accuracy, recall, precision, and F1, with higher values for better performance (Tsugawa et al., 2015; Tadesse et al., 2019). Last, we perform a multiple hold-out set validation with robust estimation. This validation provides similar quality of performance estimations to cross-validation and strikes a good balance between runtime and model validation quality (Kotu and Deshpande, 2015).

## Results

We report on the results in this section.

### Statistical results of group comparisons

Table 3 presents the means, standard deviations, and the *p*-values of the Kruskal–Wallis tests concerning the use of linguistic features in the texts of different psychological states. The results showed that the use of the four categories of linguistic features we proposed were significantly different across texts of different psychological states. That is, the linguistic features we proposed were effective in classifying texts of different psychological states.

**TABLE 3:** Statistical results of group comparisons.

		General		Anxiety		Depression		Suicide		Sig.
		Mean	SD	Mean	SD	Mean	SD	Mean	SD	
**Absolutist words**		10.69	11.26	14.98	9.90	14.74	10.12	17.69	10.71	0.00
Pronouns	Singular	51.63	46.32	84.970	26.936	85.48	28.98	97.97	28.82	0.00
	Plural	5.35	10.709	2.05	6.82	2.35	6.14	2.30	5.11	0.00
**Sentiments**		0.05	0.15	−0.13	0.15	−0.07	0.12	−0.08	0.12	0.00
Emotion	Anger	0.01	0.02	0.03	0.04	0.02	0.03	0.02	0.03	0.00
	Anticip.	0.03	0.03	0.03	0.04	0.03	0.02	0.03	0.03	0.00
	Disgust	0.01	0.02	0.02	0.02	0.02	0.02	0.02	0.02	0.00
	Fear	0.02	0.02	0.04	0.04	0.03	0.03	0.03	0.03	0.00
	Joy	0.02	0.03	0.01	0.02	0.02	0.02	0.02	0.02	0.00
	Sadness	0.02	0.02	0.04	0.04	0.03	0.03	0.04	0.03	0.00
	Surprise	0.01	0.02	0.01	0.02	0.01	0.02	0.01	0.02	0.01
	Trust	0.03	0.03	0.02	0.02	0.02	0.02	0.02	0.02	0.00

SD, standard deviation; Anticip., anticipation.

### Performance of machine-learning models

**FIGURE 1:**
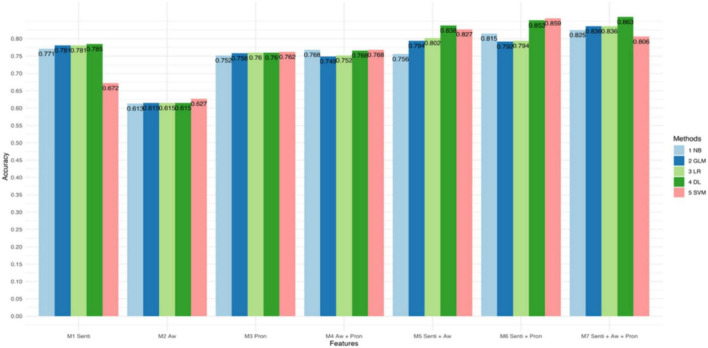
Accuracy of different models in classifying texts of anxiety.

**FIGURE 2:**
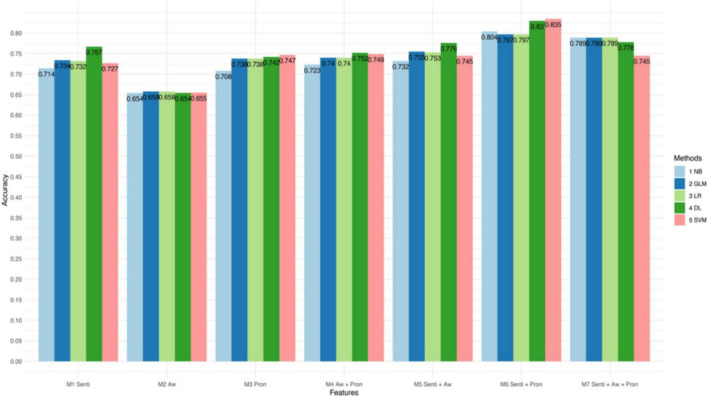
Accuracy of different models in classifying texts of depression.

**FIGURE 3:**
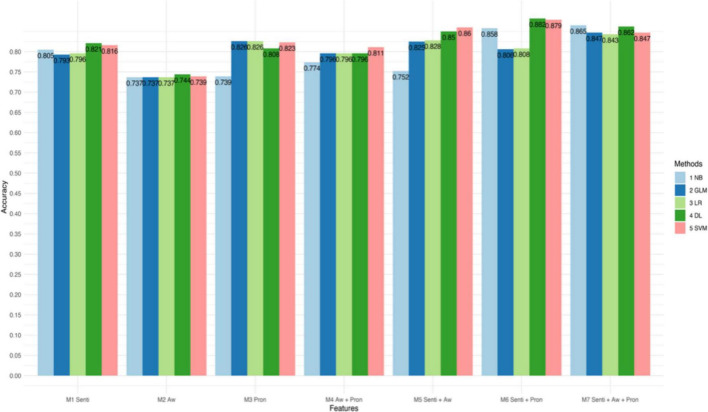
Accuracy of different models in classifying texts of suicide ideation.

Figures 1–3 and Table 4 present the performance of the linguistic features we proposed and their combinations in classifying the texts of the four psychological states. The results show some interesting findings. First, absolutist words yielded the lowest performance in detecting psychological states. Specifically, it achieved 62.7% accuracy with SVM in anxiety, 65.8% accuracy with Logistic Regression in depression, and 74.4% accuracy with Deep Learning in suicide ideation. Second, the combined linguistic features achieved the highest performance. To be specific, the combination of three types of linguistic features, i.e., personal pronouns, absolutist words, and sentiment and emotion values, produced the most accurate classification for anxiety with Deep Learning (Acc. 86.3%, Pre. 85.3%, R 94.0%, F1 89.7%). In addition, the combination of two types of linguistic features, i.e., personal pronouns and sentiment and emotion values, yielded the best classification for depression with SVM (Acc. 83.5%; Pre. 86.7%; R 88.3%; F1 87.4%) and suicide ideation with Deep Learning (Acc. 88.2%; Pre. 94.8%; R 89.0%; F1 91.8%). Third, sentiment and emotion values performed effectively in psychological state detection. For example, they achieved the best performance in the four categories of linguistic features we proposed in detecting texts of anxiety and depression (with an 86.3 and 88.2% accuracy, respectively, with Deep Learning). Also, sentiments and emotions outperformed the combination of absolutist words and personal pronouns, and when with sentiments and emotions added, the performance of the proposed linguistic features improved.

**TABLE 4:** Performance of machine-learning models.

Groups	Features	Naïve Bayes				GLM				Logistic Regression				Deep Learning				SVM			
		Acc.	Prec.	R	F1	Acc.	Prec.	R	F1	Acc.	Prec.	R	F1	Acc.	Prec.	R	F1	Acc.	Prec.	R	F1
Anxiety	Aw.	61.3	64.5	86.3	73.8	61.5	64.5	86.7	74.0	61.5	64.5	86.7	74.0	61.5	64.1	89.0	74.4	62.7	63.2	98.0	76.9
	Pron	75.2	83.4	76.0	79.4	75.8	83.7	76.7	80.0	76.0	83.8	77.0	80.2	76.0	85.6	74.7	79.7	76.2	85.9	74.7	79.8
	Senti.	77.1	78.3	88.3	83.0	78.1	75.8	96.0	84.7	78.1	75.8	96.0	84.7	78.5	76.5	95.3	84.9	67.2	65.8	100	79.4
	Aw. + Pron	76.8	77.2	90.3	83.1	74.9	77.3	85.7	81.2	75.2	77.4	86.0	81.4	76.6	79.3	85.3	82.2	76.8	79.0	86.3	82.5
	Senti. +Aw.	75.6	74.6	93.3	82.9	79.4	85.3	81.7	83.3	80.2	86.4	81.7	83.3	83.8	85.4	89.7	87.5	82.7	80.4	96.3	87.6
	Senti + Pron	81.5	88.0	82.0	84.8	79.2	92.2	73.3	81.6	79.4	92.2	73.7	81.8	85.3	93.3	82.7	87.6	85.9	94.3	82.7	88.1
	Senti + Aw. + Pron	82.5	82.3	92.3	87.0	83.6	83.5	92.3	87.7	83.6	83.3	92.7	87.7	**86.3**	**85.3**	**94.0**	**89.7**	80.6	77.4	98.0	86.5
Depression	Aw.	65.4	65.4	100	79.1	65.8	65.9	98.7	79.0	65.8	65.9	98.7	79.0	65.4	65.4	100	79.1	65.5	65.9	100	79.2
	Pron	70.8	75.4	82.3	78.7	73.8	83.4	75.0	79.0	73.8	83.4	75.0	79.0	74.2	85.0	73.7	78.9	74.7	81.6	79.3	80.4
	Senti.	71.4	78.5	78.0	78.1	73.4	74.8	89.7	81.5	73.2	74.8	89.3	81.4	76.7	76.4	93.3	84.0	72.7	71.8	96.3	82.2
	Aw. + Pron	72.3	72.8	92.0	81.3	74.0	75.8	88.7	81.7	74.0	75.8	88.7	81.7	75.2	78.4	85.7	81.8	74.9	76.3	89.7	82.4
	Senti. +Aw.	73.2	74.2	90.7	81.6	75.5	83.4	78.3	80.7	75.3	83.4	78.0	80.5	77.6	80.7	86.7	83.5	74.5	73.4	96.0	83.1
	Senti + Pron	80.4	84.1	86.7	85.3	79.7	92.8	75.0	82.8	79.7	92.1	75.7	83.0	83.0	94.8	78.3	85.7	**83.5**	**86.7**	**88.3**	**87.4**
	Senti + Aw. + Pron	78.9	77.7	95.0	85.5	78.9	79.8	91.0	85.0	78.9	79.8	91.0	85.0	77.8	76.1	96.3	85.0	74.5	72.4	98.7	83.5
Suicide	Aw.	73.7	75.5	95.3	84.2	73.7	75.5	95.3	84.2	73.7	75.5	95.3	84.2	74.4	76.4	94.7	84.5	73.9	73.9	100	85.0
	Pron	73.9	73.9	100	85.0	82.6	87.9	88.7	88.3	82.6	87.9	88.7	88.3	80.8	83.9	91.7	87.6	82.3	88.1	88.0	88.0
	Senti.	80.5	87.0	87.0	86.8	79.3	82.0	92.3	86.8	79.6	82.0	92.6	87.0	82.1	81.1	98.0	88.7	81.6	81.1	98.0	88.7
	Aw. + Pron	77.4	77.1	98.7	86.6	79.6	80.5	95.7	87.4	79.6	80.5	95.7	87.4	79.6	79.4	97.7	87.6	81.1	81.3	96.7	88.3
	Senti. +Aw.	75.2	76.3	96.3	85.1	82.5	85.2	92.3	88.6	82.8	85.5	92.3	88.8	85.0	87.5	93.0	90.1	86.0	88.6	94.7	90.9
	Senti + Pron	85.8	88.6	92.7	90.6	80.6	92.1	80.7	86.0	80.8	92.8	80.3	86.1	**88.2**	**94.8**	**89.0**	**91.8**	87.9	94.1	89.3	91.6
	Senti + Aw. + Pron	86.5	86.6	96.7	91.3	84.7	84.6	97.0	90.4	84.3	84.3	96.7	90.1	86.2	85.1	98.7	91.4	84.7	83.8	98.3	90.5

Acc., accuracy; Prec., precision; R, recall; GLM, Generalized Linear Model; SVM, Support Vector Machine; Senti., Emotion + Sentiment value; Aw., absolutist word; Pron., first-person pronouns. Best results for detection are in bold.

To summarize, the results showed that the use of the linguistic features we proposed effectively classified texts of different psychological states. The linguistic features we proposed performed best for texts of suicide ideation (with an accuracy of 88.2% on Deep Learning), and then for texts of anxiety (with an accuracy of 86.3% on Deep Learning) and for texts of depression (with an accuracy of 83.5% on SVM). In addition, the combinations that contain sentiment and emotion values improved the performance of the machine-learning models.

## Discussion

This study investigated the performance of linguistic features such as absolutist words, personal pronouns, sentiments, and emotions in identifying or classifying texts of various psychological states. The findings suggest that our proposed linguistic features, when used with machine-learning algorithms such as Support Vector Machine and Deep Learning, achieve a high level of performance for psychological state detection.

While our study included linguistic features used in previous research such as personal pronouns and absolutist words, we also experimented with additional features such as sentiment polarities and emotions (i.e., anger, anticipation, disgust, fear, joy, sadness, surprise, and trust). To the best of our knowledge, our study is probably the first attempt that uses sentiment polarities and emotions to detect texts of psychological states.

In addition, sentiment polarities and emotions were found to be effective features in detecting psychological states, and the combination of sentiment polarities and emotions with other linguistic features such as absolutist words and personal pronouns improved the performance of the machine-learning models. These findings are significant from two perspectives. On one hand, they lend evidence to the point that sentiment polarities and emotions are one of the key features for the recognition of psychological states (Desmet and Hoste, 2013; Dean and Boyd, 2020). Some possible reasons are as follows. First, expressions of sentiment polarities and emotions reveal events that people are experiencing or have experienced (Tausczik and Pennebaker, 2010). To be specific, individuals may use positive words to describe a situation that has caused positive feelings such as happiness, amusement, optimism, or satisfaction (Stirman and Pennebaker, 2001; Kahn et al., 2007). At the same time, they may use negative words to refer to an event that has caused pessimism, sadness, hopelessness, distress, deceptiveness, upheaval, or depression (Newman et al., 2003; Cohn et al., 2004; Lyons et al., 2018; Herbert et al., 2019; Jones et al., 2020). Second, a change in their psychological states may affect people’s use of sentiment and emotion words (Alvarez-Conrad et al., 2001; Newman et al., 2008). For example, people, prior to committing suicide, may use fewer positive words but more negative words that expressed or indicated negative sentiment, sadness, and/or depression (Stirman and Pennebaker, 2001; Ji et al., 2018; Kim et al., 2019). Also, compared to normal individuals, depressed ones tend to use more negative and anger words (Eichstaedt et al., 2018). Last, sentiment and emotional expressions reflect degrees of immersion (Zucco et al., 2020). That is, increased use of sentiment and emotional words may be closely related to more immersion in negative events such as traumatic experience (Tausczik and Pennebaker, 2010).

On the other hand, our study suggests that a finer-grained measure of sentiment analysis, that is, one that includes not only sentiment polarities but also subtle emotions such as anger, anticipation, disgust, fear, joy, sadness, surprise, and trust, can achieve a better performance in the detection or classification of texts showing various psychological states. Previous studies mostly used simplistic measures, such as the frequency of sentiment words, to explore the relation between sentiments and psychological states. For example, Desmet and Hoste (2013) used word frequency of 15 types of emotions to detect suicide. In this study, we achieved a higher accuracy in the classification of texts of psychological state with a finer-grained measure involving both sentiment polarities and emotions. The findings may contribute to our understanding of how to accurately detect or classify texts of various psychological states.

Furthermore, our study has confirmed that absolutist words and personal pronouns are also significant features in psychological state detection or classification. Particularly, the use of absolutist words and personal pronouns together with sentiment polarities and emotions may improve the performance in the classification of texts of psychological states. In terms of absolutist words, previous studies found that they are negatively related to mental health (Al-Mosaiwi and Johnstone, 2018). The reason is that absolutist words are associated with absolutist thinking, which is unhealthy and inflexible and may do harm to mental health (Savekar et al., 2019). Hence, people with absolutist thinking are prone to anger and self-blame (Antoniou et al., 2017), and more prone to mental disorders (Jones et al., 2020).

Personal pronouns also serve as significant features of psychological states, and the results of this study provide evidence to those of previous studies that people use more first-person singular pronouns and fewer first-person plural pronouns in sadness, anxiety, depression, suicide ideation, and suicide (Barnes et al., 2007; Wadsworth et al., 2016; Herbert et al., 2019; Kim et al., 2019). Personal pronouns measure the degree of connection and belongingness (Tausczik and Pennebaker, 2010; Allgood et al., 2020). Particularly, first-person plural pronouns may be related to social engagement, collectivism, inclusiveness, and group cohesion (Pennebaker et al., 2003), while first-person singular pronouns may be related to isolation and self-focused attention (SFA) (Sloan, 2005). It is note-worthy that the lack of belongingness seems a precursor to mental disorders such as suicide or depression (Handelman and Lester, 2007). For example, Tadesse et al. (2019) found that depressed people often feel detached and hard to integrate into society. Similarly, SFA contributes to mental disorders since it magnifies negative emotions and self-blame (Rude et al., 2004; Sloan, 2005; Kim et al., 2019).

In sum, this study verifies the value of linguistic features such as personal pronouns, absolutist words, and more importantly, sentiment polarities and emotions in the detection or classification of texts of psychological states. In addition, this study also shows the importance of machine-learning algorithms in classifying psychological states.

## Conclusion

Our study proposes an enhanced methodology that contributes to understanding the relation between linguistic features and texts of psychological states. More importantly, the current study has a significant potential for application in diverse areas. First, our effective automatic system could assist doctors or psychologists in diagnosing individuals’ mental health since it provides instant feedback with high accuracy (Wang et al., 2019). It could also be used to render support to unidentified, undiagnosed, and untreated individuals due to the stigma of mental disorders (Nadeem et al., 2016; Nguyen et al., 2017). In addition, it may be exploited to monitor social media to cope with the increasing prevalence of mental disorders (Pennebaker and Stone, 2003; Zhao et al., 2021).

Some limitations of this study should be noted. First, we should consider more factors that affect psychological health, such as social, environmental, economic, and political contexts. Second, we should consider the diachronic changes in linguistic features with mental disorders. Third, we should examine the transferable validity of our findings with more replicative studies. Future research may experiment with the proposed linguistic features, particularly sentiment polarities and emotions, on synchronous social media data such as tweets and Facebook posts for the detection or timely intervention of mental disorders.

## Data availability statement

Publicly available datasets were analyzed in this study. This data can be found here: We used Al-Mosaiwi and Johnstone’s (2018) dataset which can be accessed at https://doi.org/10.6084/m9.figshare.4743547.v1.

## Author contributions

XD created the research design, analyzed and interpreted the data, and drafted the manuscript. YS provided critical revisions. Both authors approved the final version of the manuscript for submission.

## Conflict of interest

The authors declare that the research was conducted in the absence of any commercial or financial relationships that could be construed as a potential conflict of interest.

## Publisher’s note

All claims expressed in this article are solely those of the authors and do not necessarily represent those of their affiliated organizations, or those of the publisher, the editors and the reviewers. Any product that may be evaluated in this article, or claim that may be made by its manufacturer, is not guaranteed or endorsed by the publisher.
